# Physiologic comparison between NAVA, PAV+ and PSV in critically ill patients

**DOI:** 10.1186/cc13454

**Published:** 2014-03-17

**Authors:** E Akoumianaki, G Prinianakis, E Kondili, D Georgopoulos

**Affiliations:** 1 University Hospital of Heraklion, Greece

## Introduction

The aim of the present study was to compare, in a group of difficult to wean critically ill patients, the short-term effects of PSV, PAV+ and NAVA on breathing pattern, patient effort and patient- ventilator interaction.

## Methods

Seventeen patients were studied during NAVA, PAV+ and PSV with and without artificial increase in ventilator demands (challenge) using either dead space (DS, *n *= 10) or chest elastic load (CL, *n *= 7) application. Airway and transdiaphragmatic (Pdi) pressures, electrical activity of the diaphragm (EAdi), volume and flow were measured breath by breath, while inspiratory integral of Pdi (PTPPdi) and EAdi (jEAdi) were calculated.

## Results

At resting conditions all modes provided equal support as indicated by a similar PTPPdi per breath, per minute and per liter of ventilation. Apart from triggering delay, which with and without the challenge was significantly higher with PAV+ than that with NAVA and PSV, patient-ventilatory synchrony did not differ among modes. Independent of challenging conditions, inspiratory effort to trigger the ventilator was significantly higher with PAV+ than with NAVA and PSV. Compared with PSV, PAV+ and NAVA favored a more variable breathing pattern as indicated by the significantly higher coefficient of variation of tidal volume (VT). CL increased PTPPdi significantly less with PAV+ than with PSV and NAVA, while the increase of PTPPdi after DS did not differ among modes. The relationship between VT and PTPPdi was weaker with NAVA (median (IQR) *r*^2 ^= 25.6% (2.7 to 58.1%)) than with PAV+ (55.6% (34.4 to 61.6%)) and PSV (53.9% (23.2 to 77.4%)) on account of a poor jEAdi-PTPPdi relationship (*R*^2 ^= 16.2% (1.4 to 30.9%)) during NAVA.

## Conclusion

Compared with PSV proportional modes favored breathing variability, while in the face of changing respiratory system mechanics PAV+ might be superior. However, significant drawbacks of both NAVA and PAV+ limit the effectiveness of these modes to proportionally assist the inspiratory effort.

